# Lipotoxicity and Diabetic Nephropathy: Novel Mechanistic Insights and Therapeutic Opportunities

**DOI:** 10.3390/ijms21072632

**Published:** 2020-04-10

**Authors:** Lucas Opazo-Ríos, Sebastián Mas, Gema Marín-Royo, Sergio Mezzano, Carmen Gómez-Guerrero, Juan Antonio Moreno, Jesús Egido

**Affiliations:** 1 Renal, Vascular and Diabetes Research Laboratory, IIS-Fundación Jiménez Díaz, Universidad Autónoma de Madrid, Spanish Biomedical Research Centre in Diabetes and Associated Metabolic Disorders (CIBERDEM), 28040 Madrid, Spain; lucasopazo78@gmail.com (L.O.-R.); gema.marinr@quironsalud.es (G.M.-R.); cgomez@fjd.es (C.G.-G.); jegido@quironsalud.es (J.E.); 2 Laboratorio de Nefrología, Facultad de Medicina, Universidad Austral de Chile, 5090000 Valdivia, Chile; mezzano.sergioa@gmail.com; 3 Department of Cell Biology, Physiology and Immunology, University of Cordoba, 14004 Cordoba, Spain; 4 Maimonides Biomedical Research Institute of Cordoba (IMIBIC), University of Cordoba, 14004 Cordoba, Spain; 5 Hospital Universitario Reina Sofía, 14004 Cordoba, Spain

**Keywords:** lipotoxicity, obesity, type 2 diabetes, fatty kidney, diabetic nephropathy, chronic kidney disease

## Abstract

Lipotoxicity is characterized by the ectopic accumulation of lipids in organs different from adipose tissue. Lipotoxicity is mainly associated with dysfunctional signaling and insulin resistance response in non-adipose tissue such as myocardium, pancreas, skeletal muscle, liver, and kidney. Serum lipid abnormalities and renal ectopic lipid accumulation have been associated with the development of kidney diseases, in particular diabetic nephropathy. Chronic hyperinsulinemia, often seen in type 2 diabetes, plays a crucial role in blood and liver lipid metabolism abnormalities, thus resulting in increased non-esterified fatty acids (NEFA). Excessive lipid accumulation alters cellular homeostasis and activates lipogenic and glycogenic cell-signaling pathways. Recent evidences indicate that both quantity and quality of lipids are involved in renal damage associated to lipotoxicity by activating inflammation, oxidative stress, mitochondrial dysfunction, and cell-death. The pathological effects of lipotoxicity have been observed in renal cells, thus promoting podocyte injury, tubular damage, mesangial proliferation, endothelial activation, and formation of macrophage-derived foam cells. Therefore, this review examines the recent preclinical and clinical research about the potentially harmful effects of lipids in the kidney, metabolic markers associated with these mechanisms, major signaling pathways affected, the causes of excessive lipid accumulation, and the types of lipids involved, as well as offers a comprehensive update of therapeutic strategies targeting lipotoxicity.

## 1. Introduction

Lipids are essential biomolecules for cell survival. Their role in multiple cellular functions, such as intracellular signaling, transport, immunity, maintenance of cell structure, and metabolism, highlight the importance of lipids in the regulation of cellular homeostasis [1,2,3]. Adipocytes act as fuel tanks for the storage of lipids and triglycerides. However, in non-adipocytes, which have a limited capacity to metabolize excessive lipids, their accumulation (steatosis) alter homeostasis and promote cell dysfunction [4].

Lipids accumulate in the form of lipid droplets, acting as an energy deposit for further metabolic demands [5]. The dysregulation of intracellular homeostasis as a consequence of lipids accumulation is defined as lipotoxicity, a phenomenon characterized by activation of metabolic, inflammatory, and oxidative pathways that can eventually trigger cell death [6]. Growing evidence suggests that not only the quantity of lipids but also the type of lipids accumulated may be responsible for cellular damage [7]. Throughout this review, we cover different aspects of lipotoxicity: from the causative agents and the roles and types of lipids involved in harmful effects to their potential therapeutic options, with emphasis on diabetic nephropathy (DN) and other chronic kidney diseases (CKD).

## 2. Lipotoxicity Origins

**Figure 1:**
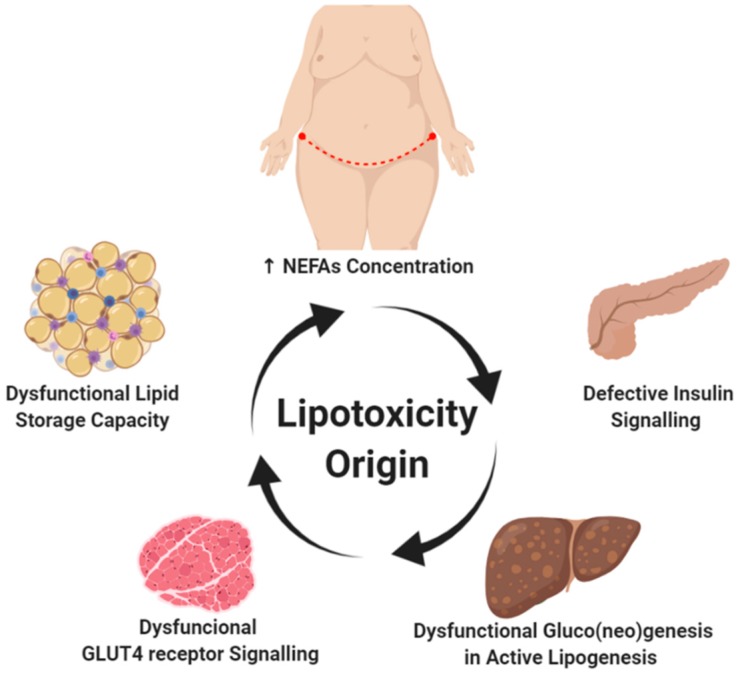
Lipotoxicity origin. The positive energy balance (high fat and/or carbohydrates diet) is one of the main promoters associated with obesity development. Hypertrophy and hyperplasia of white adipose tissue is a process commonly observed in the progression of obesity. The abdominal subcutaneous deposit has been associated with a greater increase in plasma non-esterified fatty acids (NEFA), a characteristic finding of insulin-resistant patients. Although the onset of lipotoxicity is unknown, altered lipid signaling by white adipose tissue and dysregulation in adipokines production is a key factor in restricting the lipid storage capacity observed in adipocytes. This limitation in the lipid deposit activates a vicious circle that leads to specific adaptations in energy metabolism in certain tissues such as the skeletal muscle, heart, liver, pancreas, and kidney, thus activating signaling pathways associated with gluco(neo)genesis in the presence of active lipogenesis. *Created with BioRender.com.*

Increased deposits of subcutaneous abdominal fat, high plasma concentrations of non-esterified fatty acids (NEFA), dysfunctional signaling in adipose tissue, and ectopic accumulation of lipids are closely linked to the genesis and progression of lipotoxicity (Figure 1). These causes are described in detail in this section.

### 2.1. Subcutaneous Abdominal Fatty Deposits and High Plasma NEFA Levels

Physiological lipolysis of triglycerides is a process that occurs almost exclusively in adipose tissue and its regulation depends on energy requirement [8]. NEFA are triacylglycerols stored in adipose tissue, mainly as subcutaneous abdominal fat, and transported into the plasma bound to albumin to their site of use [9]. NEFA, also called free fatty acids (FFA), are the preferential energy source of highly metabolic organs, such as the myocardium and liver. Plasma NEFA concentrations are affected by fasting state, psychological stress, and gender, among others [10,11].

An increased plasma NEFA concentration on a fasting state is related to the amount of visceral adipose tissue [12,13]. In obesity, the increase in adipose tissue seemed to correlate with plasma NEFA levels; however, a systematic review did not show an apparent correlation between the parameters [14]. Therefore, further studies will require homogenous measurements considering circadian rhythms, fasting time, and gender.

Plasma NEFA levels are also associated with type 2 diabetes (T2D). Hence, high fasting plasma NEFA concentrations are associated with glucose intolerance, regardless of the existence of previous insulin resistance or defects in insulin secretion [15,16]. Therefore, high plasma NEFA concentration is a characteristic associated with T2D and is considered an independent risk factor for insulin resistance in obese patients [17]. Indeed, in the Prospective Metabolism and Islet Cell Evaluation (PROMISE) study, total plasma NEFA concentration was a strong predictor of decreased beta-cells activity in T2D patients with one or more risk factors including obesity, hypertension, diabetes family history, gestational diabetes or neonatal high birth weight [18].

The detrimental effect of high plasma NEFA concentrations has not only been described in T2D but also in some patients with type 1 diabetes (T1D). Under physiological conditions, beta-cells can use NEFA as fuel; however, prolonged exposure to elevated levels of NEFA reduces insulin secretion by two different mechanisms, direct insulin transcriptional downregulation and beta-cell death [19,20,21]. This mechanism is not exclusive to beta-cells; it has also been observed diabetic cardiomyopathy and DN [22,23].

### 2.2. Dysfunctional Signaling in Adipose Tissue

The adipose tissue has a maximum capacity to store lipids. When this capacity is overwhelmed, adipose tissue releases lipid mediators, resulting in insulin resistance and accumulation of fatty acids in or around different organs and compartments, such as heart, pancreas, liver, blood vessels, and kidney [24].

Dyslipidemia and insulin resistance lead to a dysfunction of adipose tissue, with an increase in plasma NEFA concentrations and an imbalance between pro- and anti-inflammatory adipokines [25]. This process determines the activation of intracellular signaling pathways associated with lipid metabolism and subsequent deposition of NEFA in non-fat cells [26]. Concurrently, the microinflammatory state and the production of reactive oxygen species (ROS), through pro-oxidant enzymes such as nicotinamide adenine dinucleotide phosphate (NADPH) oxidase system, lead to the oxidative modification of lipoproteins that participate in intracellular signaling, thus promoting inflammation, oxidative stress, lipid peroxidation and vesicular transport dysfunction [27]. In this way, these events cause both endoplasmic reticulum- and lysosomal-stress altering adaptive protective mechanisms such as mitophagy, autophagy, and apoptosis, further contributing to cellular damage [28,29].

### 2.3. Insulin Resistance and Lipid Accumulation

In healthy people, insulin allows the uptake of glucose by the peripheral tissues, suppressing the production of glucose by the liver and kidney [30]. In contrast, individuals with insulin resistance have an alteration of this feedback, characterized by chronic hyperinsulinemia and slight hyperglycemia, both major factors involved in the development of diabetes [31].

Insulin resistance (IR) is a pathophysiological state caused by a gradual decline in the insulin responsiveness in peripheral tissues, including skeletal muscle, adipose tissue, and liver. The IR response and effects differ according to the affected tissue. Skeletal muscle IR is characterized by the inability to reduce blood glucose levels due to difficulty in translocating the glucose transporter type 4 (GLUT4) to the muscle cell surface membrane [32]. The defective glucose uptake results in chronic hyperinsulinemia, which activates complex lipogenic metabolic pathways, called de novo lipogenesis, resulting in the accumulation of intracellular lipids in skeletal muscle [33]. De novo lipogenesis is a highly regulated metabolic pathway whose function is to promote the synthesis of fatty acids from carbohydrates [5]. Adipose tissue IR is characterized by the inadequate lipolysis in adipocytes, whereas hepatic insulin resistance consists of decreased ability to inhibit the production of hepatic glucose in the presence of active lipogenesis [34,35,36]. Therefore, the main mechanism involved in the progression of insulin resistance is the dysfunctional signaling in adipose tissue.

## 3. Glycogen versus Lipid Storage

Studies conducted utilizing the hyperinsulinemic-euglycemic clamp technique in muscle biopsies have shown that subjects exposed to high fatty acid infusions reduce glucose oxidation, intramuscular concentration of glucose 6-phosphate, glycogen synthesis, and translocation of GLUT4 [37]. Chronic positive diet energy balance (mainly carbohydrates and saturated NEFA), increased de novo lipogenesis, and peripheral lipolysis are among the main causes favoring the ectopic accumulation of fatty acids [38,39,40].

The liver and kidney are the only organs of our body capable of producing glucose from other energy substrates such as lactate, glutamine, alanine, and glycerol, thanks to the participation of glucose 6-phosphatase, an essential enzyme to provide significant amounts of glucose into the bloodstream [41,42].

Unlike the liver, the renal glycogen deposits are essentially low. Under normoglycemic states, glucose production is provided from gluconeogenesis, specifically in the renal cortex, with an approximate contribution of 20% of the total plasma [43,44]. Under hyperglycemic conditions, a functional rearrangement of renal glucose production occurs, increasing up to three times, matching the liver supply [44]. Experimental in vitro and in vivo studies suggest the possibility to differentiate the “glucocentric and lipocentric” mechanisms involved in the progression of energy imbalance diseases [45,46]. In clinical practice, it is very difficult to differentiate the glycotoxic from lipotoxic effects, mainly due to the long exposure and synergistic interrelation of the mechanisms of action [47,48].

## 4. Dyslipidemia in Diabetic Nephropathy

Dyslipidemia is a common risk factor for cardiovascular disease, which in turn is the main cause of morbimortality in CKD and T2D patients [49]. Abnormalities in lipid and lipoprotein metabolism play a key role in the progression of renal damage and liver-mediated lipid changes are proportional to the magnitude of proteinuria, producing both quantitative and qualitative changes [50].

Obesity and metabolic syndrome are closely related to DN, the main cause of CKD worldwide [51]. In the metabolic syndrome, dysregulated lipid metabolism leads to hyperlipidemia and subsequent lipid accumulation in peripheral tissues and organs, increasing the risk for cardiovascular disease-associated death [52]. DN patients are characterized by increased plasma concentration of cholesterol, triglycerides, and apolipoprotein B (ApoB)-associated lipoproteins (very-low-density lipoprotein (VLDL), intermediate-density lipoprotein (IDL), low-density lipoprotein (LDL), and lipoprotein(a) (Lpa)), along with decreased levels of high-density lipoproteins (HDL) (ApoA-I) [53,54]. Additionally, these changes are accompanied by incrementing other apolipoprotein subtypes, such asApoB100 (involved in lipoprotein uptake), ApoB48 (the main component of chylomicrons), and ApoC-III (lipoprotein lipase inhibitor) [50,54]. In the hyperglycemic, inflammatory, and oxidizing context of DN, these irregularities in lipid metabolism promote atherogenicity and progression of kidney damage [55].

Several mechanisms are implicated in the abnormal biosynthesis, transport, and clearance of lipids and lipoproteins in DN. Thus, DN patients show reduced expression of lipoprotein lipase, disrupted reverse cholesterol transport, and decreased the number of receptors mediating lipids uptake [56].

Along with the quantitative changes, DN patients show important qualitative changes in lipoprotein composition. Indeed, HDL particles show enrichment in triglycerides and loss of anti-oxidants, such as paraoxonase [56,57]. In T2D patients, urinary albumin creatinine ratio directly associates with small LDL particle concentrations, whereas estimated glomerular filtration rates are inversely associated with small VLDL and medium HDL particles [58]. Moreover, increased levels of oxidized HDL and LDL have been detected in the plasma and renal parenchyma of patients with CKD, and are more abundant in kidneys from hyperlipidemic animals [59,60,61]. These atherogenic lipoproteins are cytotoxic and may affect the behavior or renal cells, contributing to the progression of renal damage [62,63].

Injurious actions of oxidized lipoproteins include tubular apoptosis and oxidative stress by increasing NADPH-oxidase mediated ROS production, recruitment of circulating monocytes, and increased production/secretion of pro-inflammatory cytokines by tubular cells, such as IL-6, CCL2, CCL5, and TNF-alpha [64,65,66].

The relation between circulating NEFA and hypertriglyceridemia is reciprocal as lipotoxicity leads to triglyceride secretion and sustained hypertriglyceridemia leads to adipose dysfunction. The latter is another factor involved in the CKD-mediated lipotoxicity [4]. In DN, white adipose tissue is reduced and cytotoxic NEFA is not stored in this tissue. Consequently, NEFA remains in plasma for more time, increasing fat accumulation in other tissues [67].

## 5. The Fatty Kidney in DN

In the kidney, ectopic lipid deposition contributes to the local inflammation and oxidative stress [30,68]. In DN patients, dyslipidemia promotes ectopic lipid accumulation and lipid intermediates (e.g., palmitate, ceramides, and saturated NEFA), not only in kidney but also in extra-renal tissues such as liver, pancreas, and heart [4,49,69].

**Figure 2:**
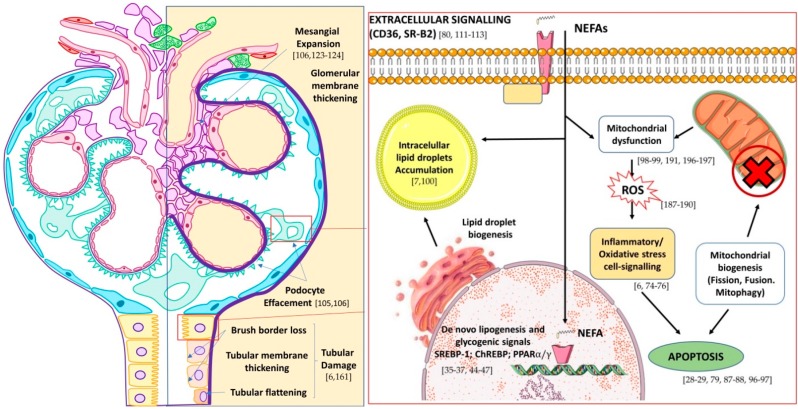
Effect of lipotoxicity on kidney nephron (**left**) and main pathways of action and detoxification of non-esterified fatty acids (NEFA) in podocytes and tubular cells (**right**). In brackets are shown selected references on lipotoxicity-mediated mechanisms. Adapted from Wikimedia glomerule image (CC BY-SA 4.0 Author: M. Komorniczak).

The fatty kidney condition has been widely described in the literature [70,71,72,73,74]. This pathological condition is characterized by lipid accumulation in the renal parenchyma causing damage to various cells, including podocytes, proximal tubular epithelial cells, and the tubulointerstitial tissue through various mechanisms, being potentially detrimental to renal function in the long term [70,71,72,73,74]. On the other hand, DN patients usually have albuminuria, which is a well-known risk factor for the progression of renal disease [51,61]. In addition to its direct toxic effect, albumin may act as a carrier of fatty acids in urine. Therefore, albuminuria may favor a massive accumulation of fatty acids in the kidney, thus promoting tubular damage in DN patients [75]. In this sense, it has been demonstrated that albumin-bound fatty acid, but not albumin itself, promotes oxidative stress and apoptosis in tubular cells [76]. Other proteins, such as the protein cluster of differentiation 36/fatty acid translocase (CD36/FAT) and fatty acid transport protein 2 (FATP2), facilitate the tubular toxicity of fatty acids by increasing their uptake and metabolism [60,66]. Besides, induction of ATF6α, a transcription factor of the unfolded protein response, enhanced lipid accumulation and apoptosis in tubular cells (Figure 2) [77].

Renal steatosis is associated with lipid droplets in different intracellular compartments, as noticed in transmission electron microscopy or magnetic resonance imaging studies. Lipids droplets may act as lipid precursors that participate in energy metabolism, intracellular signaling and vesicular transport [3]. The presence of lipid droplets in renal cells is not exclusive to patients with pathologies associated with obesity and diabetes [78]. Cytoplasmatic lipid droplets accumulation influences the progression of inflammation and fibrosis in several genetic and metabolic pathologies [79]. These lipid mediators have been associated with the activation of the inflammatory response, ROS production, mitochondrial dysfunction, autophagy deregulation, endoplasmic reticulum stress (ER stress) and apoptosis [69,80,81]. This fact is mainly due to the accumulation of intermediary toxic metabolites, such as diacylglycerol, fatty acyl-CoA, ceramides, and sphingolipids, which are involved in protein kinase C (PKC) activation, triglyceride synthesis, and mitochondrial dysfunction [82,83].

### Lipotoxicity in Renal Cells

The lipotoxicity effects in kidney are variable according to the cell type involved. The renal lipid accumulation in human DN and age-related renal disease is mainly concentrated in the tubule although glomerular deposits are also noticed associated with glomerular hypertrophy and tubulointerstitial fibrosis [7,84]. Among the affected glomerular cells, specific damage has been described in podocytes, mesangial, and endothelial cells and macrophages [85,86].

**Podocytes:** The relationship between lipids and podocyte damage is well known. Indeed, apolipoprotein-1 (APOL1), an essential component of HDL3, is highly expressed in podocytes. APOL1 is the highest risk genetic variant of kidney disease and the main cause of glomerulosclerosis in the African-American population [87]. On the other hand, phospholipase A2 (PLA2), an enzyme that hydrolyzes lipids to release arachidonic acid and other inflammatory lipid mediators, is closely linked to the development of nephrotic syndrome and promotes kidney injury via its M-subtype receptor [88].

The loss of podocytes and retraction of the foot processes are involved in the development of renal injury in DN [89]. Increased podocyte detachment has been observed in patients with obesity related-glomerulopathy, focal segmental glomerulosclerosis (FSGS), and DN [90]. In this regard, podocytopenia could be mediated by alterations in the lipid balance [90,91].

The sterol regulatory element-binding proteins (SREBPs) are the main transcription factors associated with lipid metabolism through their direct role in renal lipid accumulation [84]. Upregulated SREBP expression is found in lipid droplets loaded podocytes, both in experimental studies and in a retrospective analysis of renal biopsies of patients with DN or obesity-related glomerulopathy [7,91,92]. Intracellular lipid is imbalanced towards accumulation when lipid efflux is impaired.

Excessive cholesterol accumulation and cholesterol efflux is downregulated in experimental and human DN [7,93,94]. ATP-binding cassette transporter A1 (ABCA1), G1 (ABCG1), and scavenger receptor class B type I (SR-BI), present at podocytes, mesangial cells, macrophages, and proximal tubular cells, are the main cholesterol efflux transporters studied in DN [95,96,97]. Growing evidence has validated their renoprotective effects due largely to the modulation of receptors associated with cholesterol efflux [97,98,99,100]. Merscher-Gomez et al. showed that human podocytes exposed to serum from DN patients, increased the presence of cell plasma membrane blebbing, lipid droplets and reduced the expression of the ATP-binding cassette A1 (ABCA1), a scavenger receptor associated with cholesterol efflux, whereas no changes were observed after stimulation with serum from diabetic patients without DN and healthy controls. Downregulation of ABCA1 expression was noted in kidney biopsies of DN patients [101,102]. Furthermore, the authors proposed a renoprotective action of 2-hydroxypropyl-β-cyclodextrin by preventing renal cortex cholesterol accumulation mediated by ABCA1 upregulation [101,103].

In this regard, ABCA1 deficiency has been associated with the selective accumulation of cardiolipin, mitochondrial dysfunction, and subsequent podocyte lesion in DN [104]. Mitrofanova et al. described the involvement of the lipid raft enzyme sphingomyelinase-like phosphodiesterase 3b (SMPDL3b) in podocyte injury by altering active sphingolipid production and reducing ceramide-1-phosphate (C1P) [104,105]. Furthermore, restoration of C1P levels was sufficient to normalize the proteinuria levels observed in a murine model of DN [106].

**Mesangial cells:** Mesangial cell expansion is a histological feature usually seen in patients with T2D or obesity-associated kidney damage and precedes glomerulosclerosis evolution in DN [90,107]. Initial stages of the renal damage associated with obesity or pre-diabetes are characterized by the presence of albuminuria, glomerular hypertrophy, and mesangial expansion [108]. Human mesangial cells express LDL receptors and CD36/FAT scavenger receptors, being the endothelial glycocalyx dysfunction especially sensitive for cholesterol, and modified lipoproteins accumulation [70]. Additionally, mesangial cells can also accumulate triglycerides by the lipoprotein lipase action [109].

De novo lipogenesis mediated by SREBP-1 has been described as a new mediator of TGF-β1 signaling pathway, thus its activation plays a key role in the progression of inflammation and glomerular fibrosis in this cell type under diabetic conditions [110,111,112].

Indeed, lipotoxicity, in general, and exposure to NEFA, in particular, directly affect mesangial cell functions by activating the intrinsic apoptosis pathway, although the mechanisms of toxicity are not fully elucidated [113,114,115,116]. Furthermore, the use of fenofibrate (PPARα agonist) can prevent lipid-induced toxicity and oxidative stress in glomerular cells by inducing the expression of lipolytic enzymes [117].

**Endothelial cells:** Although endothelial cells do not seem to be prone to lipid accumulation, their role is essential in the transport of lipids to other tissues, particularly in the renal area, thus they should be the main source of lipid supply to glomerular cells [118]. This process has been described to be mediated by the co-expression of VEGF-B and mitochondrial proteins, and therefore to be a therapeutic target against the mechanisms of lipotoxicity at systemic level [119,120,121].

**Macrophages/Foam cells:** The role of macrophages and foam cells in kidney lipotoxicity is dual. First, lipid accumulation in renal cells promotes macrophages recruitment [122]. Second, lipotoxicity directly activates macrophages, which results in their transdifferentiation [123,124,125]. Excessive and chronic uptake of lipids by macrophages is an aggravating factor involved in the progression of glomerular injury and atherosclerosis in CKD patients [126,127].

Foam cells are specialized scavenger cells, which are mainly derived from monocytes/macrophages, although renal cells (mesangial, epithelial, smooth muscle, and endothelial cells) may also be involved in foam cells formation [127,128,129]. Foam cells remove modified lipoproteins (oxidized LDL, acetylated-LDL, and β-VLDL) [130,131]. This uptake is mediated by the expression of scavenger receptors and LDL receptors on their surface [127].

Although foam cells are related to atherosclerosis progression, preclinical studies in kidneys biopsies from FSGS and DN. FSGS and DN patients showed increased accumulation of foam cells and the presence of intracellular lipid droplets [132,133].

Several studies have demonstrated the importance of the inflammatory milieu in the foam cell formation [91,134,135]. Cytokines such as TNFα and IL-1β can modify LDL receptor-mediated cholesterol uptake, by increasing SREBP translocation and promoting foam cells formation in macrophages and mesangial cells [134,136].

Scavenger receptor A (SR-A) and class B-2 scavenger receptor (SR-B2), specifically CD36/FAT, are the main NEFA-uptake mediator in both physiological and pathophysiological situations [137]. These scavenger receptors are key elements in the fatty acid uptake, expressed in podocytes, mesangial cells, endothelial cells, and tubular cells, which is currently proposed as a potential therapeutic target in preclinical kidney disease [115,138,139,140]. It has recently been described that the presence of advance oxidation protein products (AOPP) and hyperglycemia lead to CD36/FAT upregulation, as well as to increased CD36-dependent signaling through Wnt/β-catenin and PKC pathways, which is associated in the epithelial tubular cells with oxidative stress, inflammation, and fibrosis development [139,141,142].

**Proximal tubular cells:** Proximal tubules are also damaged as a consequence of lipotoxicity, mainly in proteinuric kidney diseases such as nephrotic syndrome [50]. Tubular uptake of NEFA is directly proportional to the detected proteinuric levels and basolateral NEFA concentration [70]. Intracellular lipid accumulation is associated with the phenomena of tubular flattening, loss of brush border, and tubulointerstitial fibrosis [6,143].

From a physiological point of view, plasma NEFA are almost entirely bound to albumin and only a very small percentage (<0.01%) is free [9]. Because the glomerular filtration barrier prevents the passage of albumin, the low percentage that can be incorporated into the ultrafiltrate is fully reabsorbed due to receptor-mediated endocytosis [144]. If there is damage in the glomerular filtration barrier, then the proximal tubule will reabsorb almost all filtered albumin-bound NEFA [145]. After the incorporation of albumin into the cytosol, this protein is degraded into amino acids by lysosomal activity, while NEFA freely diffuses until it can be compartmentalized in lipids droplets [145]. This phenomenon has been described as the “trojan horse effect”, allowing NEFA incorporation by tubular cells [6]. Trojan horse effect can be exacerbated by an increase in albumin filtration (alterations in glomerular basement barrier) and/or increase in the molar ratio free fatty acid/albumin (e.g., dyslipidemia) [76,145,146].

Katsoulieris et al. observed lipotoxic effects on tubular cells with physiological levels of saturated fatty acids such as palmitic acid by downregulation of stearoyl-CoA desaturase-1 (SCD-1), key regulator of lipotoxicity-induced damage [147,148]. Studies in vitro demonstrated that SCD-1 overexpression ameliorated fatty acid-mediated cell toxicity, manifesting a possible protective role in experimental podocytes and tubular damage [149,150]. Another signal potentially involved in lipotoxicity-mediated tubular damage is the AMPK-PGC-1α pathway. As this pathway activity decreases, an increase in the intra-cytoplasmic triglyceride synthesis and accumulation is observed [151]. PGC-1α, a key regulator of mitochondrial biogenesis and energy metabolism, is proposed as one of the main therapeutic targets in acute kidney damage, CKD, and DN [152,153,154,155,156,157]. In this sense, preservation of the mitochondrial cristae structure with Elamipretide (D-Arg-2′,6′-dimethyl-TyrLys-Phe-NH2) restores AMPK activity and prevents glomerulopathy and intracellular lipid accumulation in the proximal tubule in high-fat diet model [158]. These findings confirm that reduced lipotoxicity optimizes kidney function and prevents its progression, both at glomerular and tubulointerstitial level.

## 6. Mitochondria as the Main Target of Lipotoxicity-Kidney Disease

Alterations in mitochondria function determine the intracellular accumulation of lipids and further lipotoxic actions, as reported in both glomeruli and tubules [159,160,161]. The proximal tubule has highly specialized metabolic machinery, which needs a fine energy balance. In DN, the maintenance of insulin resistance state affects functionally at the mitochondrial level, and depending on the energy requirements, the incorporated fatty acids can be metabolized by β-oxidation [156].

The suppression of β-oxidation described in the DN and advanced CKD patients could be one of the mechanisms that exacerbate the accumulation of lipid drops at the mitochondrial level [161,162,163]. This event turns out to promote the loss of cristae structure, with degeneration and mitochondrial swelling, preventing optimal energetic functioning in different renal cell types [159]. Structural mitochondrial integrity is necessary for the proper functioning of the oxidative phosphorylation and production of ATP [164]. Continuous oxidation of glucose at the mitochondrial level is a highly regulated enzymatic process, which allows the formation of energy necessary for cellular viability [156].

Recent studies have highlighted the renoprotective role of cardiolipin, a unique phospholipid present in the mitochondrial inner membrane, necessary for the formation and maintenance of mitochondrial cristae structure [158,159,165]. The prevention of cardiolipin peroxidation improves the efficiency of ATP production through the oxidative phosphorylation (OXPHOS) process, restoring mitochondrial bioenergetics [160,161]. Indeed, when phosphorylation capacity is exceeded, mitochondria are disturbed and, as a consequence, non-oxidized lipids are accumulated. This mitochondrial vicious cycle could be a main process of lipotoxicity in kidney disease [156,161,166,167]. These intermediate molecules promote insulin-resistance, activate mitophagy and promote mitochondrial fission or fusion as well as dysregulation of the cell cycle [167].

Mitochondrial fission or fusion maintain mitochondrial morphology and adequate energy activity [164]. While the fusion allows the incorporation of specific enzymes and/or mitochondrial DNA to improve and repair functions, fission attempts to isolate defective proteins for subsequent autophagy recycling, generating healthy mitochondria [168]. Although the mechanisms of renal damage caused by lipotoxicity are different in the glomerular and tubulointerstitial compartment, it is widely known that its action is multifactorial, since the involvement of one compartment directly affects the other [169]. In the same way, mitophagy is another specialized mechanism of autophagy used in the case of dysfunction and/or energy deficit [166]. In DN, an increase in mitochondrial fission and fragmentation via mitophagy has been observed in proximal tubules [157].

Recent studies have proposed a mitochondrial “hormetic” hypothesis in the progression of DN, which attempts to reflect that continuous production of ROS would be a “healthy mitochondria” indicator [170,171]. The persistence of these injury mechanisms would initiate a gradual reduction in normal mitochondrial oxidative capacity, leading to a depletion of enzyme complexes and subunits necessary for the free radicals production [156]. This proposal modifies the renal energy paradigm, observing a significant increase in the production of mitochondrial ROS during the initial phases of DN, and then decreasing jointly with the reduction of renal function [169,170,171].

The Warburg effect, a hypothesis widely used to describe the cancer cells metabolism, would explain the mitochondrial dysfunction observed in the final stage of patients with diabetic kidney disease [172].

## 7. Lipid Biomarkers as Predictors of DN

The use of omics, specifically lipidomics and MALDI-TOF mass spectrometry, has allowed the characterization of lipid abnormalities in the study of complex metabolic disorders, such as diabetes and its complications [173,174]. Searching for novel associations between serum lipidomics and DN has been quite fruitful, as shown in Table 1. Thus, Tofte et al. found that sphingomyelin and phosphatidylcholine species were associated with lower risk progression of renal impairment and all-cause mortality in T1D individuals [175].

Other authors have identified urine and plasma lipid metabolites as new biomarkers of DN development in patients with T2D [176,177,178]. Recently, an integrative transcriptomic-lipidomic analysis has identified lipid mediators (unsaturated NEFA, phosphatidylethanolamines, short-low double bond triacylglycerols, and long-chain acylcarnitines) as predictors of the progression of diabetic kidney disease in American Indians with preserved renal function (GFR ≥ 90 mL/min) [162].

The changes in the lipid profile have also been studied. In general, a decrease in plasma EFAs and an increase in NEFA have been noted [176]. In other metabolomic studies, molecules related to the transport of lipids into the mitochondria, such as acylcarnitine (e.g., butenoylcarnitine) or involved in carnitine synthesis (e.g., γ-butyrobetaine), have consistently been shown elevated, probably indicating a decreased fatty acid oxidation/mitochondrial dysfunction and maybe early lipotoxicity [177,178].

## 8. Targeting Lipotoxicity in DN. Is This Approach Feasible?

**Figure 3:**
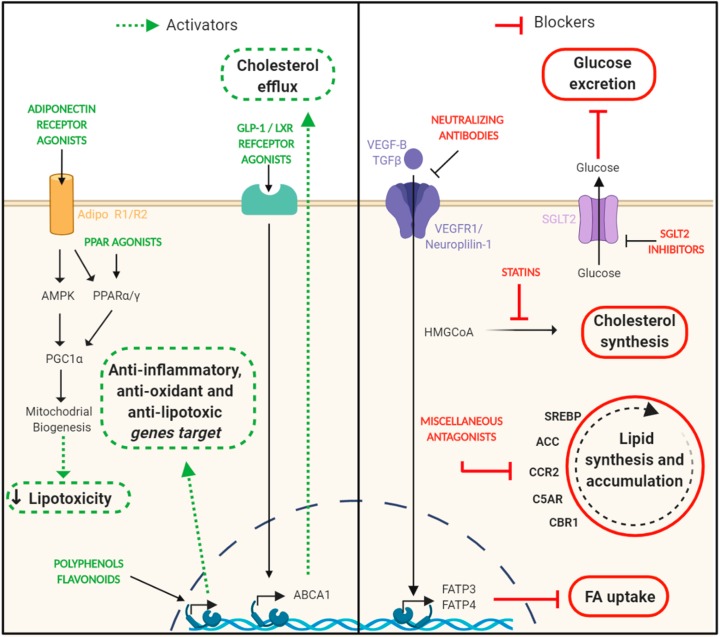
Targeting lipotoxicity in DN. The intrarenal lipids reduction has been positively correlated with renoprotective effects observed in the progression of experimental diabetic nephropathy. On the left side, there are represented different strategies focused on enhancing signaling pathways that are considered beneficial to reduce lipid accumulation (adiponectin/PPAR signaling and cholesterol efflux) and prevent other derived damages such as inflammation and oxidative stress. On the right side are shown different approaches focused on blocking signaling pathways that have a harmful effect in the context of diabetic nephropathy (glucose excretion, cholesterol synthesis, lipid synthesis, and accumulation and FA uptake). ABCA1, ATP-Binding Cassette Transporter A1; ACC, acetyl-CoA carboxylase; AMPK, AMP-activated protein kinase; CBR1, Carbonyl reductase 1; CCR2, C-C chemokine receptor type 2; C5AR, complement component 5a receptor 1; FA, fatty acid; FATP3/4, fatty acid transport protein 3/4; GLP-1, glucagon-like peptide 1; LXR, live X receptor; PGC1α, peroxisome proliferator-activated receptor gamma coactivator 1-alpha; PPARα/γ, peroxisome proliferator-activated receptors α/γ; SGLT2, sodium-glucose co-transporter 2 inhibitors; SREBP, sterol regulatory element-binding proteins; TGFβ, transforming growth factor-beta; VEGF-B, vascular endothelial growth factor-beta; VEGFR1, vascular endothelial growth factor receptor. *Created with BioRender.com.*

Interventions targeting lipotoxicity are of course better exemplified on fatty liver disease and its clinical consequences. Lifestyle modifications including healthy eating and regular exercise are the primary recommendations [179,180]. Pharmacological interventions are now being evaluated in clinical trials in those patients. Treatments include drugs targeting energy intake, energy disposal, lipotoxic liver injury, and the resulting inflammation, fibrogenesis, and cirrhosis. In this context, several drugs are undergoing phase 2/3 trials in nonalcoholic steatohepatitis [181]. Besides, the potential beneficial effects of drugs with anti-lipotoxic effects in other organs, including the kidney, are still unknown. Several compounds have been examined in experimental preclinical DN models, demonstrating renoprotection and reduction of kidney lipid accumulation (Figure 3).

In this regard, there are no proper clinical trials with those drugs in patients with DN. Herein, we briefly summarize some observational studies suggesting that some anti-lipotoxic drugs could also be effective in diabetic patients with CKD, as well as preclinical evidence reporting renoprotective effects and reduced lipid accumulation in several experimental models of DN (Table 2).

### 8.1. Statins

Statins are currently the main lipid-lowering drugs. Its mechanism of action is based on the inhibition of 3-hydroxy-3-methyl-glutaryl-coenzyme A (HMG-CoA) reductase, an enzyme involved in cholesterol synthesis [182]. This inhibition reduces not only plasma LDL-cholesterol levels but also triglycerides, thus preventing pathological effects of oxidized LDL-induced injury and cell signaling activation in the glomerular compartment [183]. Beyond the lipid-lowering potential, statins have anti-inflammatory, anti-oxidant, and anti-proliferative properties, and also protect the endothelium and increase adiponectin levels [184]. Tonolo et al. demonstrated that simvastatin reduced urinary albumin excretion rate in hypertensive and normotensive T2D patients [185]. They also support the hypothesis that the improvement in renal function is related to a reduction in oxidative stress, thus limiting glomerular damage [186]. In favor of this hypothesis, mevastatin, pravastatin, and simvastatin are also able to prevent apoptosis and loss of nephrin induced by oxidized LDL in cultured podocytes [183].

A recent meta-analysis demonstrated that, although statins lowered proteinuria and all-cause mortality, this effect was not sufficient to slow the clinical progression of non-end-stage CKD [187]. Despite these findings, all available clinical guides suggest that controlling LDL-cholesterol is a part of the multi-target approach in the treatment of DN [188].

### 8.2. PPAR Agonists

Considering that some of the beneficial effects of statins are due to their ability to interact with PPARs, it is not surprising the development of PPAR therapies in the context of obesity and diabetes [20]. Thiazolidinediones (TZDs), synthetic ligands to PPARγ, were thought to be potential favorable candidates to treat DN. However, adverse effects associated with TZD treatment, such as bone loss, fluid retention, and cardiovascular complications, among others, have limited their clinical use for the treatment of DN [221].

In studies on mesangial cells and an experimental T2D model of leptin receptor-deficient mice (db/db), Hong et al. showed that fenofibrate improved albuminuria, inhibited intrarenal NEFA and triglyceride accumulation, and prevented apoptosis and oxidative stress [154]. In mice fed with a high-fat diet, fenofibrate also reduced oxidative stress and lipid accumulation in glomeruli and prevented the development of albuminuria and glomerular fibrosis [117]. Moreover, PPARα/γ dual agonist Tesaglitazar, not only improved lipid metabolism and increased adiponectin levels, but also prevented albuminuria and renal glomerular fibrosis in diabetic mice [222]. These results highlight dual PPARα/γ agonists as a potential therapy to treat renal lipotoxicity and diabetic kidney disease.

### 8.3. Adiponectin Receptor Agonists

Adiponectin is an adipokine secreted by adipocytes and involved in fatty acid metabolism. It has been demonstrated that adiponectin is decreased in the context of T2D and metabolic syndrome, thus leading to fatty acids accumulation [223]. Adiponectin exerts its activity through the binding with its receptors, AdipoR1 and AdipoR2, and further activation of PGC-1α via the phosphorylation of AMPK or PPARγ activation, respectively [224,225]. The activation of the AMPK-PPARγ-PGC-1α axis decreases lipid levels in the bloodstream as well as in ectopic tissues, such as the kidney. Therefore, adiponectin has emerged as an important target against lipotoxicity-mediated harmful effects [226]. Different therapeutic approaches are under development aiming to increase adiponectin serum levels or adiponectin receptor expression/activity [227].

Thus, recent studies have shown the beneficial effect of AdipoRon, an orally-active synthetic adiponectin agonist, which was able to reduce lipotoxicity and to improve insulin resistance, obesity-related disease, and DN and its complications [227,228,229,230]. AdipoRon reduced sphingolipids levels, which are closely related to lipotoxicity, inflammation and insulin resistance [189]. Moreover, AdipoRon ameliorated renal alterations in db/db mice by reducing oxidative stress and apoptosis in the kidney [190].

### 8.4. SGLT-2 Inhibitors

SGLT2 inhibitors (SGLT2i) are new drugs for the treatment of patients with T2D and its complications and have contributed to open a new era in cardiorenal protective medicine [231,232,233,234]. Recent findings from observational and randomized controlled trials have also revealed that SGLT2i can decrease the fatty liver content, as assessed by different imaging techniques, and improve biological markers of NAFLD, especially serum liver enzymes, in patients with T2D [233]. In one of these studies, Kahl et al. showed that empagliflozin effectively reduces hepatic fat in patients with T2D with excellent glycemic control and short known disease duration [235]. In another observational study, including 59 patients with T2D, the oral fixed-dose combination of the SGLT2i dapagliflozin and dipeptidyl peptidase-4 inhibitor (DPP4i) saxagliptin significantly decreased liver fat and adipose tissue volume versus glimepiride, and reduced serum liver enzyme levels, indicating a favorable metabolic profile of this combination in patients with T2D on metformin therapy [236]. In a larger study, in 695 patients with T2D, exenatide and dapagliflozin combination improved markers of liver steatosis and fibrosis [237].

The potential beneficial effect of SGLT2i on the removal of fatty deposits in other organs such as the heart or the kidney remains unexplored, at least in patients. However, at the experimental level, some studies have been reported. Recently, Hosokawa et al. showed that Ipragliflozin decreases ectopic lipid accumulation in tubular cells in diabetic mice [238]. In *db/db* mice, JNJ 39933673, a selective SGLT2i, prevented renal lipid accumulation by inhibition of transcription factor carbohydrate-responsive element-binding protein (ChREBP) β-isoform, a transcription factor that mediates activation of several regulatory enzymes of glycolysis and lipogenesis pathway such as SCD-1 and diacylglycerol O-acyltransferase-1 (DGAT1) [191]. Although further studies are needed overall, it is plausible that, in addition to the well-known effects of SGLT2i (anti-inflammatory, anti-proliferative, and anti-fibrotic), the reduction of tubular lipid deposition could be a new renoprotective mechanism of these molecules [192,239,240]. In this context, Exendin-4 and Liraglutide, two GLP-1 receptor agonists, ameliorated obesity-induced chronic kidney injury by modulating AMPK-SIRT1-PGC-1α pathway and enhancing ABCA1-cholesterol efflux [193,194].

### 8.5. VEGF-B Signaling inhibition

Vascular endothelial growth factor B (VEGF-B) has been described as one of the major responsible for lipid control in endothelial cells [118,119]. VEGF-B, through its union with receptors located on the cell surface as VEGFR1 and Neuropilin-1, induces the expression of the fatty acid transport proteins FATP3 and FATP4, favoring lipid accumulation [121]. Modulation of VEGF-B signaling prevented insulin resistance and dyslipidemia, reducing lipid accumulation in podocytes [120]. Renoprotective effects have been observed with the administration of neutralizing VEGF-B antibodies in T1D and T2D mice, mainly regulating lipid accumulation in podocytes [120,195].

### 8.6. Polyphenols, Flavonoids, and Nutraceuticals

Polyphenols, flavonoids, and food rich-flavonoids also present lipid-lowering properties that could ameliorate lipotoxicity in diabetic kidney disease [241]. Recently, a study performed by Jayachandran et al. demonstrated the capacity of isoquecertin to regulate lipid metabolism via AMPK pathway [196]. Besides, quercetin was also able to reduce lipid accumulation in the kidney, individually or in association with allopurinol, a uric acid inhibitor [197,198]. Resveratrol and anthocyanin-rich Seoritae extract prevents lipotoxic and glucotoxic effects through AMPK-PGC-1α axis in db/db mice [199,200]. The anti-lipotoxic effect of curcumin, berberine, oligonol-derived lychee fruit, and oryzanol-derived rice bran oil has also been demonstrated due to their capacity to reduce inflammatory, oxidative stress, and mitochondrial dysfunction markers in diabetic murine models [201,202,203,204,205]. Tangshen formula, a traditional Chinese formulation, was able to alleviate abnormal renal lipid accumulation and kidney damage in db/db mice by promoting ABCA1-mediated efflux cholesterol [206]. Thymol, a monoterpene phenolic compound found mainly in oil of thyme (an herb known as *Thymus vulgaris*) and Omacor (*n*-3 polyunsaturated fatty acids), decreased renal lipid accumulation through the modulation of SREBP-1-mediated lipogenic pathway [207,208]. Although further studies are needed, these antioxidant molecules could potentially be an effective therapy against lipotoxicity-mediated kidney injury.

### 8.7. Other Drugs Able to Impair Renal Lipid Deposition

To restrict intrarenal lipid deposition, novel therapeutic targets have been evaluated in different preclinical models, such as ATP-binding cassette transporter A1 (ABCA1) agonists [96,99,104], renal lipoprotein lipase activators [209], Farnesoid X receptor (FXR) [210] and Liver X receptor alpha (LXRα) agonists [211], pan-TGFβ neutralizing antibodies [212,213], NF-κB inhibitors [214], fibroblast growth factor-21 therapy [215], aspirin [216], angiotensin 1–7 [217], CCR2 inhibitors [218], C5a receptor antagonists [219], cannabinoid receptor-1 blockers [220], and Nrf2 activators such as the bardoxolone methyl [171].

### 8.8. Non-Pharmacological Approaches

The beneficial effect of nutritional restriction and subsequent weight loss is a clear demonstration of the role that lipotoxicity plays on T2D development and progression [242]. In the DiRECT study, intensive nutritional intervention ameliorated T2D by resorption of ectopic lipid accumulation, particularly the pancreatic ones, was considered of paramount importance [243,244,245,246].

Regular exercise is one of the fundamental pillars for bodyweight reduction and decrease local and systemic inflammatory microenvironment [247,248,249]. Increasing energy demand by skeletal muscle contraction promotes glucose homeostasis by peripheral lipolysis and liver NEFA oxidation [250,251,252]. In addition to bodyweight reduction (with its anti-proteinuric effect), the improvement of metabolic kidney milieu and endothelial function restoration could also account for the renoprotective effects of regular exercise on diabetic chronic disease [253,254,255].

Furthermore, in patients with morbid obesity, bariatric surgery may have, similar to other forms of severe weight loss, additional renoprotective effects besides the well-known metabolic control [134,254,255]. The effect of reducing lipid accumulation in NAFLD constitutes at present a subject of paramount importance with several ongoing clinical trials, but, to date, there are no such studies on kidney diseases.

## 9. Perspectives and Conclusions

Lipotoxicity is a common finding observed in metabolic diseases. The kidney is a target-organ of lipotoxicity-mediated harmful effects, principally associated with obesity, insulin resistance, plasma NEFA increase, and adipose tissue dysfunction. The dramatic increase in the prevalence of obesity has been accompanied by a series of comorbidities including the development of T2D, cardiovascular disease, and non-alcoholic fatty liver disease [180].

Overall, in this review, we examine the recent preclinical and clinical research about the potentially harmful effects and causes of excessive lipid accumulation in the kidney as well as the types of lipids involved. Targeting kidney lipotoxicity with novel drugs addressed to treat non-alcoholic fatty liver disease could potentially constitute an additional alternative to combat the complex mechanisms implicated in diabetic nephropathy and lipotoxicity kidney disease.

## Figures and Tables

**Table 1:** Selected articles on lipotoxicity-related biomarkers in diabetic nephropathy. Lipid metabolites are labeled in bold.

Condition	Patients	Sample	Comparison	Disease-Associated Metabolites	Ref.
**T2D**	92 (American Indians)	Serum	DN Progression	**↑Polyunsaturated triacylglycerols (TAGs)** **↓C16–C20 acylcarnitines (ACs)**	[163]
**T1D**	669	Serum	Combined renal end-point	**↓PC(O-34:2), PC(O-34:3), SM(d18:1/24:0), SM(d40:1), SM(d41:1).**	[175]
			All-cause mortality	**↓PC(O-34:3), SM(d40:1) and SM(d41:1).**	
			Albuminuria progression	**↓SM(d18:1/24:0)**	
**T2D + renal involvement**	150	Plasma	Control-T2D	**↓EFAs** **↑NEFAs**	[176]
			T2D-DN III	**↑EFAs** **=NEFAs**	
			DN III-DN IV	**↓EFAs** **↓↓NEFAs**	
			DN IV-DN V	**↑EFAs** **↑NEFAs**	
**T2D**	90	Plasma	Δ UACR or Δ eGFR	↓Histidine; **↑butenoylcarnitine**	[177]
		Urine		↓Hexose, Glutamine, Tyrosine	
**T2D**	78	Serum	Albuminuria	↑Creatinine, aspartic acid, γ-butyrobetaine, citrulline, symmetric dimethylarginine (SDMA), kynurenine, azelaic acid, galactaric acid	[178]

Abbreviations: TAGs, Triacylglycerols; ACs, Acylcarnitines; PC; Phosphatidylcholine; SM; Sphingomyelin; EFA, Esterified fatty acids; NEFA, Non-esterified fatty acids; UACR, Urinary albumin, creatinine ratio; eGFR, estimated glomerular filtration rate. DN III, IV, and V refer to stages in diabetic nephropathy development.

**Table 2:** Summary of selected preclinical studies reporting renoprotective effects and reduction of kidney lipid accumulation.

Drug	Category	Pathway	Experimental Model	Observed Effect	Ref.
**Cyclosporin A2/hydroxypropyl-β-cyclodextrin**	Calcineurin inhibitor/lipid chelator	TNF/NFAT/ABCA1/SOAT1 signaling	Podo-*Abca1* KO, double, triple, and inducible KO mice	↓ UACR; ↓ Histological changes; ↓ Inflammation/oxidative stress/apoptosis; ↓ Lipid accumulation	[96]
**A30/Elamipretide**	ABCA1inductor/Cardiolipin peroxidase inhibitor	Mitochondrial dysfunction pathway	Podo-*Abca1* KO SOAT KO, *db/db and* BTBR *ob/ob* mice	↓ UACR; ↓ Histological changes; ↓ Oxidative stress/Mitochondrial dysfunction; ↓ Lipid accumulation	[104]
**Ceramide-1-Phosphate**	Lipid supplementation	SMPDL3b/C1P/IR/Cav-1/Akt signaling	Podo-*Smpdl3 KO,* double KO, *and db/db* mice	↓ UACR; ↓ Histological changes; ↓ Lipid accumulation	[106]
**Fenofibrate**	Fibrate	PPARα modulator	HFD	↓ Albuminuria; ↓ Histological changes; ↓ Oxidative stress/fibrosis; ↓ Lipid accumulation	[117]
**Fenofibrate**	Fibrate	PPARα modulator/AMPK-PGC-1α axis	*db/db* mice	↓ Albuminuria; ↓ Histological changes; ↓ Inflammation/oxidative stress; ↓ Apoptosis/fibrosis; ↓ Lipid accumulation	[154]
**AdipoRon**	Adiponectin agonist	AMPK/PPARα pathway	*db/db* mice	↓UACR; ↓ Oxidative stress/apoptosis/fibrosis; ↓ Lipid accumulation	[189,190]
**Ipraglifozin**	SGLT2i	ER stress pathway	FTL *ob/ob* mice	↓ Histological changes; ↓ ER stress/apoptosis/fibrosis; ↓ Lipid accumulation	[191]
**JNJ-39933673**	SGLT2i	Glycogenic and lipogenic pathways	*db/db* mice	↓ UACR; ↓ Histological changes; ↓ Inflammation/fibrosis; ↓ Lipid accumulation	[192]
**Exendin-4**	GLP-1 RA	Cholesterol efflux pathway	ApoE KO HFD + STZ	↓UACR; ↓ Lipid accumulation	[193]
**Liraglutide**	GLP-1 RA	AMPK/SIRT1/PGC-1α axis	SD rats + HFD	↓UACR; ↓ Inflammation/fibrosis; ↓ Lipid accumulation	[194]
**Neutralizing Monoclonal VEGF-B antibody**	VEGF-B antagonism	VEGF-B signaling	Podo-*VegfB*KO; *db/db* mice; double KO; + HFD; + STZ	↓ UACR; ↓ Histological changes; ↓ Inflammation; ↓ Lipid accumulation	[195]
**Isoquercetin**	Flavonoid	NF-κB-AMPK-NRF2 axis	Wistar rats + STZ	↓ Inflammation/oxidative stress; ↓ Lipid accumulation	[196]
**Quercetin**	Flavonoid	SCAP-SREBP-2-LDLR pathway	*db/db* mice	↓UACR; ↓ Histological changes; ↓ Lipid accumulation	[197]
**Quercetin + Allopurinol**	Flavonoid + uric acid inhibitor	NLRP3 Inflammasome pathway	SD rats + STZ	↓ Albuminuria; ↓ Histological changes; ↓ Inflammation; ↓ Lipid accumulation	[198]
**Anthocyanin-rich Seoritae extract**	Flavonoid	AMPK/PGC-1α	*db/db* mice	↓ Albuminuria; ↓ Oxidative stress; ↓ Apoptosis/fibrosis; ↓ Lipid accumulation	[199]
**Resveratrol**	Polyphenol	AMPK–SIRT1–PGC-1α axis	*db/db* mice	↓ Albuminuria; ↓ Histological changes; ↓ Inflammation/oxidative stress; ↓Apoptosis/fibrosis; ↓ Lipid accumulation	[200]
**Curcumin**	Polyphenol	AMPK/NRF2 pathway	OLETF rats	↓ Albuminuria; ↓ Histological changes; ↓ Inflammation/oxidative stress; ↓ Lipid accumulation	[201]
**Curcumin**	Polyphenol	AMPK/SREBP-1 pathway	SD rats + STZ	↓ Albuminuria; ↓ Histological changes; ↓ Inflammation/fibrosis; ↓ Lipid accumulation	[202]
**Oligonol-derived lychee fruit**	Polyphenol	Adiponectin pathway	*db/db* mice	↓ Histological changes; ↓ Inflammation ↓ Apoptosis/oxidative stress; ↓ Lipid accumulation	[203]
**Oryzanol Concentrate**	Rice bran oil	SREBP-1 pathway	Wistar rats + HFD + STZ	↓ Albuminuria; ↓ Histological changes; ↓ Inflammation/oxidative stress/fibrosis; ↓ Lipid accumulation	[204]
**Berberine**	Flavonoid	Mitochondrial dysfunction pathway	*db/db* mice	↓ UACR; ↓ Histological changes; ↓ Oxidative stress; ↓ Mitochondrial dysfunction; ↓ Lipid accumulation	[205]
**Tangshen Formula**	Traditional Chinese formulation	PGC-1α-LXR-ABCA1 pathway	*db/db* mice	↓ UACR; ↓ Histological changes; ↓ Lipid accumulation	[206]
**Thymol**	Monoterpene phenolic compound	SREBP-1 pathway	HFD	↓ Albuminuria; ↓ Histological changes; ↓ Oxidative stress/fibrosis; ↓Lipid accumulation	[207]
**Omacor**	*n*-3 polyunsaturated fatty acid	NF-κB and lipogenic pathway	*db/db* mice	↓ Albuminuria; ↓ Histological changes; ↓ Inflammation/fibrosis; ↓ Lipid accumulation	[208]
**Ibrolipim (NO-1886)**	Renal lipoprotein lipase agonist	Activation renal lipoprotein lipase	CB minipigs + HSFD	↓ UACR; ↓ Histological changes; ↓ Lipid accumulation	[209]
**Obeticholic acid**	FXR agonist	Glutathione metabolism pathway	HFD + UNX	↓ UACR; ↓ Histological changes; ↓ Oxidative stress/apoptosis; ↓ Lipid accumulation	[210]
**GW3965**	LXRα agonist	LXRαin macrophages	LDLR KO and transgenic mice; WD + STZ	↓ Albuminuria; ↓ Histological changes; ↓ Inflammation/oxidative stress/fibrosis; ↓ Lipid accumulation	[211]
**1D11**	pan-TGFβ-neutralizing antibody	TGFβ-ApoB axis	Double KO STZ + CholD	↓ UACR; ↓ Fibrosis; ↓ Lipid accumulation	[212,213]
**Celastrol**	NF-κB inhibitor	NF-κB pathway	*db/db* mice	↓ UACR; ↓ Histological changes; ↓ Inflammation/oxidative stress/fibrosis; ↓ Lipid accumulation	[214]
**Fibroblast growth factor-21**	Growth factor	TGFβ pathway	FGF21 KO mice + STZ; BSA–FFA	↓ UACR; ↓ Inflammation/oxidative stress/apoptosis; ↓ Lipid accumulation	[215]
**Aspirin**	COX-2 inhibitor	COX-2/LDLR pathway	SD rats + STZ	↓UACR; ↓ Histological changes; ↓ Inflammation; ↓ Lipid accumulation	[216]
**Angiotensin 1–7**	ACEi	ACE2/Ang 1–7/Mas receptor axis	*db/db* mice	↓ Albuminuria; ↓ Histological changes; ↓ Inflammation/oxidative stress/fibrosis; ↓ Lipid accumulation	[217]
**RS504393**	CCR2 antagonist	CCL2/CCR2 axis	*db/db* mice	↓ Albuminuria; ↓ Histological changes; ↓ Inflammation/fibrosis; ↓ Lipid accumulation	[218]
**NOX-D21**	Complement C5a inhibitor	C5a/C5a receptor axis	*db/db* mice + UNX	↓ UACR; ↓ Histological changes; ↓ Inflammation/fibrosis; ↓ Lipid accumulation	[219]
**SR141716**	CB-1 receptor antagonist	CB-1 receptor pathway	*db/db* mice	↓ UACR; ↓ Histological changes; ↓ Inflammation/oxidative stress/fibrosis; ↓ Lipid accumulation	[220]

Abbreviations: FXR, Farnesoid X receptor; GLP-1 RA, Glucagon-like peptide-1 receptor agonist; TGFβ, Transforming growth factor beta; LXRα, Liver X receptor alpha; SGLT2i, Sodium-glucose cotransporter 2 inhibitors; NF-κB, Nuclear factor kappa B; ACEi, Angiotensin-converting-enzyme inhibitor; CCR2, C-C chemokine receptor type 2; CB-1, Cannabinoid 1 receptor; VEGF-B, Vascular endothelial growth factor B; ABCA1, ATP binding cassette A1; COX-2, Cyclooxygenase 2; PPARα, Peroxisome proliferator-activated receptor alpha; AMPK, AMP-activated protein kinase; SREBP, Sterol regulatory element-binding protein; ApoB, Apolipoprotein B; PGC-1α, Peroxisome proliferator activated receptor-gamma coactivator-1; SCAP, SREBP cleavage-activating protein; Bgn, Biglycan; LDLR, Low-density lipoprotein receptor; SIRT1, Sirtuin-1; ACE2, Angiotensin I converting enzyme 2; Ang 1–7, Angiotensin 1–7; CCL2, C-C motif chemokine ligand 2; TNF, Tumor necrosis factor; NFAT, Nuclear factor of activated T-cells; SOAT1, Sterol O-acyltransferase 1; SMPDL3b, Sphingomyelinphosphodiesterase acid-like 3b; C1P, Ceramide-1-phosphate; IR, Insulin receptor; Cav-1, Caveolin-1; Akt, Protein kinase B; NRF2, Nuclear factor erythroid 2-related factor 2; NLRP3, NOD-, LRR- and pyrin domain-containing protein 3; HFD, High-fat diet; STZ. Streptozotocin; CholD, Cholesterol diet; WD, Western diet; ApoE, Apolipoprotein E; BSA, Bovine serum albumin; FFA, Free fatty acid; TG, transgenic; ob/ob, leptin-deficient; db/db, Leptin receptor-deficient; podo, Podocyte; fl/fl, FloxingCre-Lox recombination mice; BTBR, Black tan and brachyury; HSFD, High sugar fat diet; UACR, Urinary albumin creatinine ratio.
